# Efficient and accurate determination of the degree of substitution of cellulose acetate using ATR-FTIR spectroscopy and machine learning

**DOI:** 10.1038/s41598-025-86378-0

**Published:** 2025-01-23

**Authors:** Frank Rhein, Timo Sehn, Michael A. R. Meier

**Affiliations:** 1 Institute of Mechanical Process Engineering and Mechanics (MVM), Karlsruhe Institute of Technology (KIT), Karlsruhe, 76131 Germany; 2 Institute of Biological and Chemical Systems – Functional Molecular Systems (IBCS-FMS), Karlsruhe Institute of Technology (KIT), Karlsruhe, 76344 Germany; 3 Institute of Organic Chemistry (IOC), Karlsruhe Institute of Technology (KIT), Karlsruhe, 76131 Germany

**Keywords:** Machine learning, Degree of substitution, Infrared spectroscopy, Cellulose ester, Cellulose acetate, Chemistry, Analytical chemistry, Cheminformatics, Green chemistry, Organic chemistry

## Abstract

Multiple linear regression models were trained to predict the degree of substitution (DS) of cellulose acetate based on raw infrared (IR) spectroscopic data. A repeated k-fold cross validation ensured unbiased assessment of model accuracy. Using the DS obtained from ^1^H NMR data as reference, the machine learning model achieved a mean absolute error (MAE) of 0.069 in DS on test data, demonstrating higher accuracy compared to the manual evaluation based on peak integration. Limiting the model to physically relevant areas unexpectedly showed the ${\hbox {C}{-}\hbox {H}}$ peak to be the strongest predictor of DS. By applying a n-best feature selection algorithm based on the F-statistic of the Pearson correlation coefficient, several relevant areas were identified and the optimized model achieved an improved MAE of 0.052. Predicting the DS of other cellulose acetate data sets yielded similar accuracy, demonstrating that the developed models are robust and suitable for efficient and accurate routine evaluations. The model solely trained on cellulose acetate was further able to predict the DS of other cellulose esters with an accuracy of $\approx 0.1-0.2$ in DS and model architectures for a more general analysis of cellulose esters were proposed.

## Introduction

Fossil resource depletion combined with the increased pollution of our environment are the main reasons for a change towards biobased polymeric materials. Cellulose, as the most abundant biopolymer on earth ($1.5\times 10^{12}$ tons p.a.)^1,2^, plays a key role in establishing biobased alternatives to fossil based polymers due to its unique properties. Besides biocompatibility and biodegradability, cellulose also possesses high thermal and mechanical resistance, both being important requirements for potential industrial applications^3,4^. Up to the present day, cellulose esters (CEs) are the most important cellulose derivatives from an industrial point of view^5^. The synthesis of CEs can be performed according to several synthetic procedures in a homogeneous^1,4,6–8^ or heterogeneous fashion^9–12^. The material properties and processability of CEs depend on the applied synthesis procedures as well as on their structural composition, i.e., the nature of the ester and the degree of substitution ($\text{DS}$)^6,13^. The $\text{DS}$ is defined as the average number of substituted hydroxyl groups per anhydroglucose (AGU) unit and can adopt values between 0 and 3. CEs find applications in coatings, drug delivery, food packaging, membrane and fiber industry, or bio-medicine^3,4,14,15^. Especially cellulose acetate (CA), which is most commonly known as the material used for cigarette filters, can be recognized as the CE with the highest industrial interest so far^5^.

The material properties of CA can be adjusted by varying the $\text{DS}$. Thus, it is essential to have reliable, simple and fast analytical tools for the determination of the $\text{DS}$ of CAs and other CEs. The most common method for $\text{DS}$ determination, which is also recognized as the most time- and labor-intensive one, is the hydrolysis of CEs in an alkaline medium and subsequent titration^8,16^. Additionally, this method suffers from a large sample demand and is also categorized as a destructive analysis method. A more straightforward non-destructive alternative for $\text{DS}$ determination of CEs based on nuclear magnetic resonance spectroscopy (NMR) are ^1^H- and ^13^C-NMR^1,14,15,17–20^. These approaches are limited by the solubility of the CEs in solvents suitable for NMR analysis. This is especially challenging for CEs with low $\text{DS}$, and requires the synthesis of more elaborate solvent systems and adjusted NMR protocols^21^. A supplemental requirement for accurate $\text{DS}$ determination via ^1^H- and ^13^C-NMR is that the magnetic resonances of the introduced substituents do not overlap with the signals of the anhydro glucuse units (AGU). King et al.^22^ introduced a more advanced technique that involves the derivatization of unmodified hydroxyl groups with a phosphorous reagent and a subsequent quantitative ^31^P NMR evaluation. Other analytical techniques, such as gas chromatography^23^, elemental analysis^24,25^ or UV–Vis spectroscopy^26^ are reported, but limited with respect to precision and applicability. Wolfs et al.^27^ reported a supplemental and more convenient $\text{DS}$ determination for CEs via attenuated total reflection Fourier transform infrared (ATR-FTIR) spectroscopy by simple integration of the ${\hbox {C}{=}\hbox {O}}$, ${\hbox {C}{-}\hbox {O}}_{\text{ester}}$, and ${\hbox {C}{-}\hbox {H}}$ stretching vibration bands. After fitting of calibration curves, $\text{DS}$ values could be determined with an average relative error between 5.5 % and 7.3 % depending on the evaluated peaks. These values are similar to the reported accuracy of the ^1^H NMR method and—compared to the labor-intensive NMR-based characterization methods—this approach does not require elaborate sample preparation and measurements are very fast ($\approx 30\,$s). Nevertheless, as this $\text{DS}$ determination via ATR-FTIR still requires the manual selection and integration of the corresponding peaks in the spectra, this study raises the question whether the procedure can be automated, streamlined and/or enhanced by applying machine learning techniques.

Machine learning (ML) is a branch of artificial intelligence that aims at inferring solutions to problems by applying statistical methods to data instead of explicitly programming the solution. Regression is a subset of ML tasks and describes the prediction of dependent variables (outcomes) based on independent variables (inputs, features). Different algorithms can be used to achieve this, including multiple linear regression (MLR) or neural networks (NN). The adjustment (optimization) of internal parameters to the specific data set is referred to as learning and the obtained parameter set in conjunction with the algorithm is considered a model. If learning is based on provided, static data of input-output pairs, this is referred to as supervised learning.

ML, although currently an omnipresent term, is by no means a new idea and has been tied to spectroscopy from the very beginning. It can be argued that one of the earliest examples is the well-known work of Beer^28^, who—although without computers at the time—fitted extinction coefficients of a linear model to experimental observations. More contemporary examples, actually based on algorithmic learning without human evaluation, are often summarized under the term chemometrics^29^ and are applied to various spectroscopic measurements^30^, like Raman spectroscopy^31,32^, UV/Vis absorption spectroscopy^33,34^, NMR spectroscopy^35,36^, or IR spectroscopy^37,38^. The combination of IR and regression analysis was applied to determine the degree of oxidation of dialdehyde cellulose^39^ and the $\text{DS}$ of both carboxymethyl starch^40^ and methylesterified pectic polysaccharides^41^. The $\text{DS}$ of CA specifically, was accurate determined by regression analysis of FT Raman spectra^42^. Further, ML was applied to determine the cellulose and lignin content of lignocellulose originating from different sources with high accuracy^43^. ML techniques applied to NIR measurements were able to accurately predict cellulose pulp dryness in real time^44^, and the hemicellulose, cellulose and lignin content of moso bamboo^45^.

The aim of this work is to find accurate ML models for predicting the $\text{DS}$ of CA based on raw IR spectra, which has not been reported before. Such a model would be fast and not require any manual data processing or evaluation, two properties that are highly relevant for quality control in industry and unbiased analytical procedures in academia. Multiple linear regression models are trained, while a repeated k-fold cross-validation allows for an unbiased evaluation of model accuracy. The results are compared to the values obtained from ^31^P NMR, ^1^H NMR and the integration-based method proposed by Wolfs et al.^27^ to assess accuracy. However, this study is not solely concerned with predicting $\text{DS}$ values. Features (wavenumbers) are selected based on physical knowledge and algorithms to reveal the most relevant areas of the spectra, i.e. areas that contain most information. Finally, an optimized model is applied to CA data of different experimenters and different synthesis routes as well as to IR spectra of other CEs to quantify the robustness and extrapolation capabilities of the model. This has not been reported before and paves the way for more generalized analytical models.

## Data overview

All data sets used in this study were obtained by us in our laboratory and have been previously published^1,6,27^. Table 1 provides an overview of the different data sets with corresponding reference, number of data points *N*, $\text{DS}$-range and additional details. Data sets are marked with monospace font and named after the first author (e.g. Wolfs.A). All data sets contain ^1^H NMR spectroscopy and IR spectroscopy data. The reference set Wolfs.A additionally contains ^31^P NMR spectroscopy data for $\text{DS}>1.21$ (insolubility below). $\text{DS}$ values were determined in various ways: $\mathrm {DS_{1H}}$ and $\mathrm {DS_{31P}}$ are obtained by applying the routine described in the methods section and^22^ to ^1^H NMR and ^31^P NMR data, respectively. $\mathrm {DS_{IR,int}}$ is obtained via peak integration of IR data and corresponds to $\mathrm {DS_{IR,mean}}$ of^27^. $\mathrm {DS_{IR,ML}}$ is obtained by the ML evaluation methods proposed in this study. More information on the sample preparation and analyses is found in the supporting information for this article or the respective publications. All compiled data is provided in open access^46^.

**Table 1:** Data overview.

Name	Refs.	*N*	$\mathrm {DS_{1H}}$-range	Details
Wolfs.A	^27^	16	$0.41-2.96$	Cellulose acetate, reference data set^a^.
Wolfs.B	^1^	5	$1.04-2.5$	Cellulose acetate, same chemistry^a^, partially different experimenter
Sehn.A	^6^	7	$0.80-2.34$	Cellulose acetate, different chemistry^b^, different experimenter
Sehn.B	^6^	5	$2.05-2.17$	Cellulose esters of different chain lengths with $\text{DS}\approx 2$, different chemistry^b^, different experimenter
Sehn.C	^6^	5	$1.99-2.16$	Cellulose esters of different chain lengths (mixtures) with $\text{DS}\approx 2$, different chemistry^b^, different experimenter

$^{\text{a}}$ Modification of MCC with vinyl acetate in a DMSO/DBU/CO_2_ switchable solvent system at $60\,^{\circ }$C (conventional heating) within $6\,$h (see supporting information).

$^{\text{b}}$ Modification of MCC with vinyl acetate in a DMSO/TMG/CO_2_ switchable solvent system at $140\,^{\circ }$C (microwave heating) within $10\,$min (see supporting information)

## Results and discussion

### ^31^P NMR versus ^1^H NMR versus IR integration accuracy based on data set Wolfs.A

**Fig. 1:**
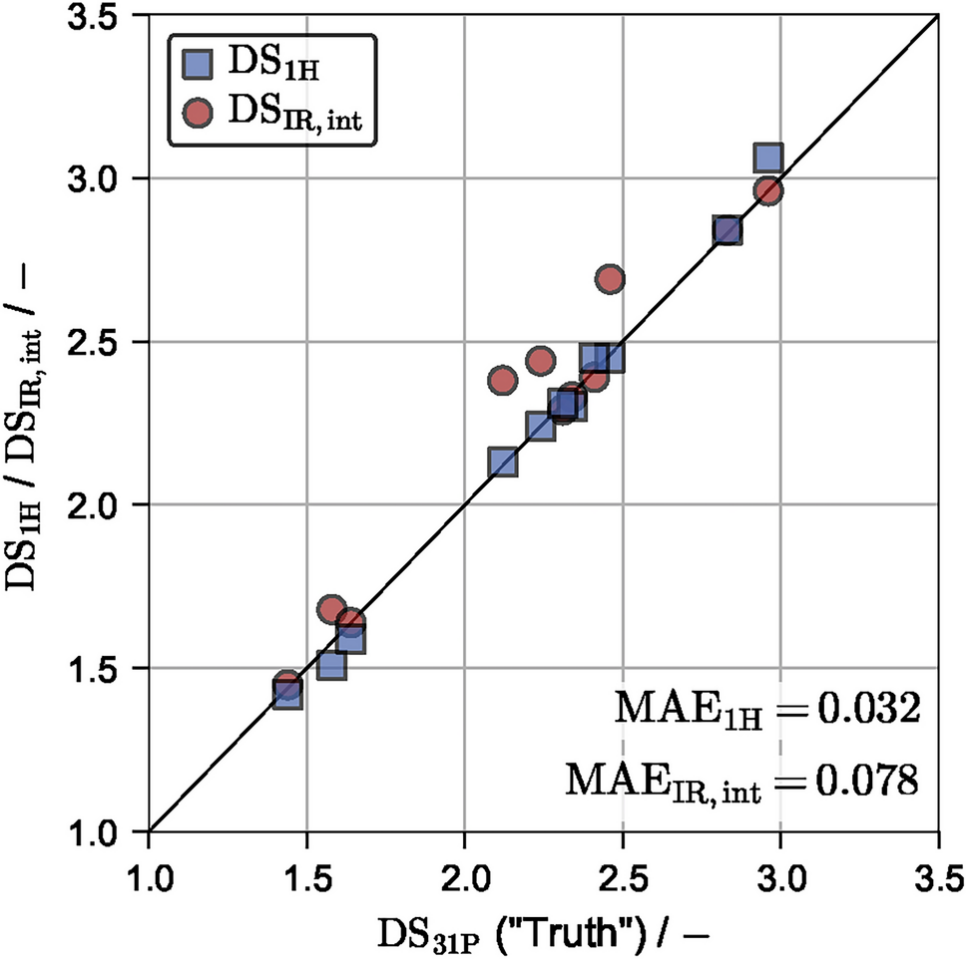
$\mathrm {DS_{31P}}$ values versus $\mathrm {DS_{1H}}$ and $\mathrm {DS_{IR,int}}$ for the Wolfs.A data set. Only $\text{DS}>1.21$ values are considered due to insolubility of the samples below this value.

The obtained $\mathrm {DS_{1H}}$ values based on the described integration routine and reported $\mathrm {DS_{IR,int}}$ values are compared to the reported $\mathrm {DS_{31P}}$ values of Wolfs.A. It is assumed that ^31^P NMR provides the most accurate $\text{DS}$ values. Please note that only data points with $\text{DS}>1.21$ can be considered due to insolubility of the samples below this $\text{DS}$ value. ^1^H NMR data achieved a better $\text{MAE}$ of 0.032 ($\text{MRE}=1.53\%$), while IR integration reported previously showed a $\text{MAE}$ of 0.078 ($\text{MRE}=3.64\%$). The results are visualized in Fig. 1.

To extend the study also to $\text{DS}$ values smaller than 1.21, all following investigations are performed with $\mathrm {DS_{1H}}$ values as ground truth, i.e. as training data. Based on Fig. 1 and considering that ^31^P NMR data is also susceptible to experimental errors, this assumption appears justified.

**Fig. 2:**
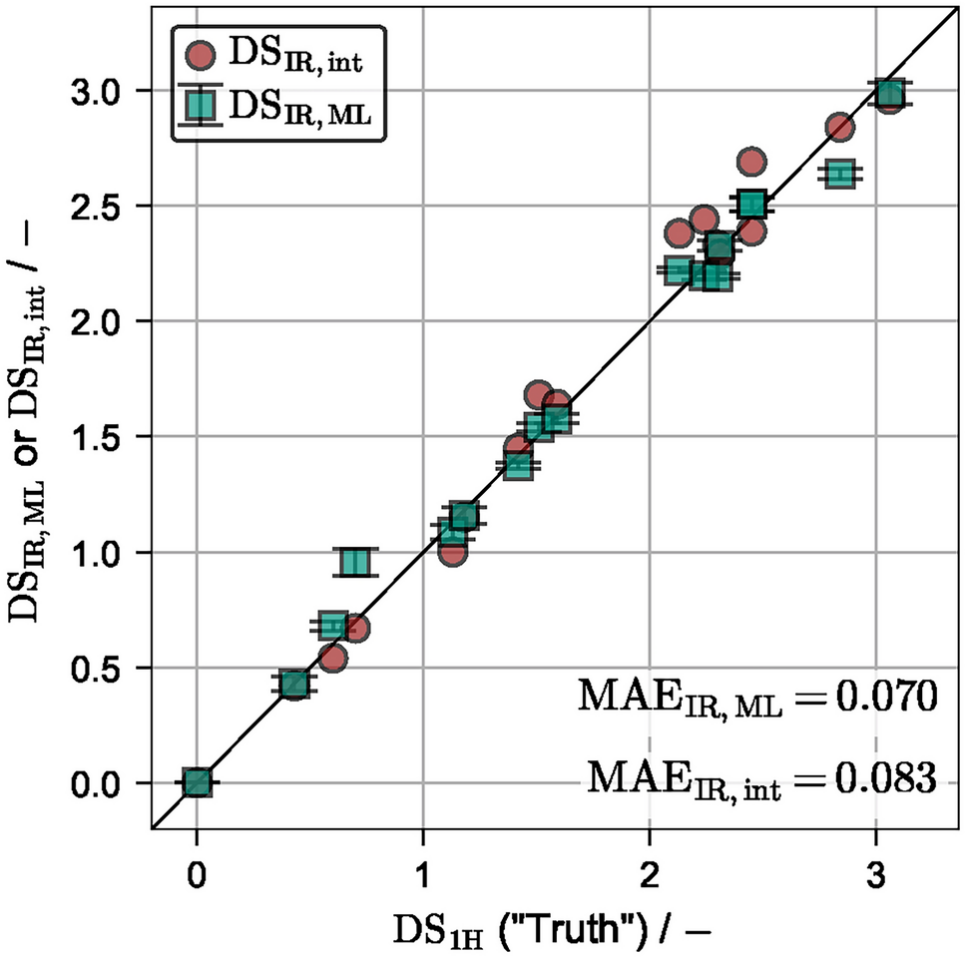
$\mathrm {DS_{1H}}$ vs. $\mathrm {DS_{IR,ML}}$ and $\mathrm {DS_{IR,int}}$ values for the Wolfs.A data set. The mean values and corresponding standard deviation of all test data points from *N* repetitions and *k* folds are shown.

### ML prediciton accuracy on data set Wolfs.A

#### Baseline accuracy

A repeated *k*-fold cross validation (CV, see methods section) was performed with randomized data assignment between repetitions on the $\mathrm {DS_{1H}}$ data of Wolfs.A. A multiple linear regression (MLR) model is used with $N=1000$ repetitions and $k=8$ folds, meaning that roughly $12.5\,\%$ of the data points were reserved for testing in each iteration. The results are shown in Fig. 2. A $\mathrm {MAE_{IR,ML}}$ of 0.069 ($\mathrm {MRE_{IR,ML}}=5.73\%$) is obtained. Additionally, the $\mathrm {DS_{IR,int}}$ values are plotted against the $\mathrm {DS_{1H}}$ values. A $\mathrm {MAE_{IR,int}}$ of 0.083 ($\mathrm {MRE_{IR,int}}=5.34\%$) is obtained. Hence, the ML evaluation achieved a slightly smaller $\text{MAE}$ and slightly larger $\text{MRE}$ than the manual IR integration method, although both evaluation methods are precise and achieve $\text{MAE}<0.1$. This immediately demonstrates that a simple linear regression model is able to make good predictions on the $\text{DS}$ based on provided raw IR spectra. It should be highlighted that only the prediction accuracy on the test data, i.e. unseen data that has not been used in training, is shown, while for IR integration data, no test/train split was performed. It is apparent that the integration method consistently overpredicts the $\text{DS}$ values, while the ML evaluation appears less biased. The opposing behavior in $\text{MAE}$ and $\text{MRE}$ can be explained by the large prediction error of the ML model at the relatively small $\mathrm {DS_{1H}}$ value of 0.70, resulting in a larger relative error.

#### Influence of k-fold

**Fig. 3:**
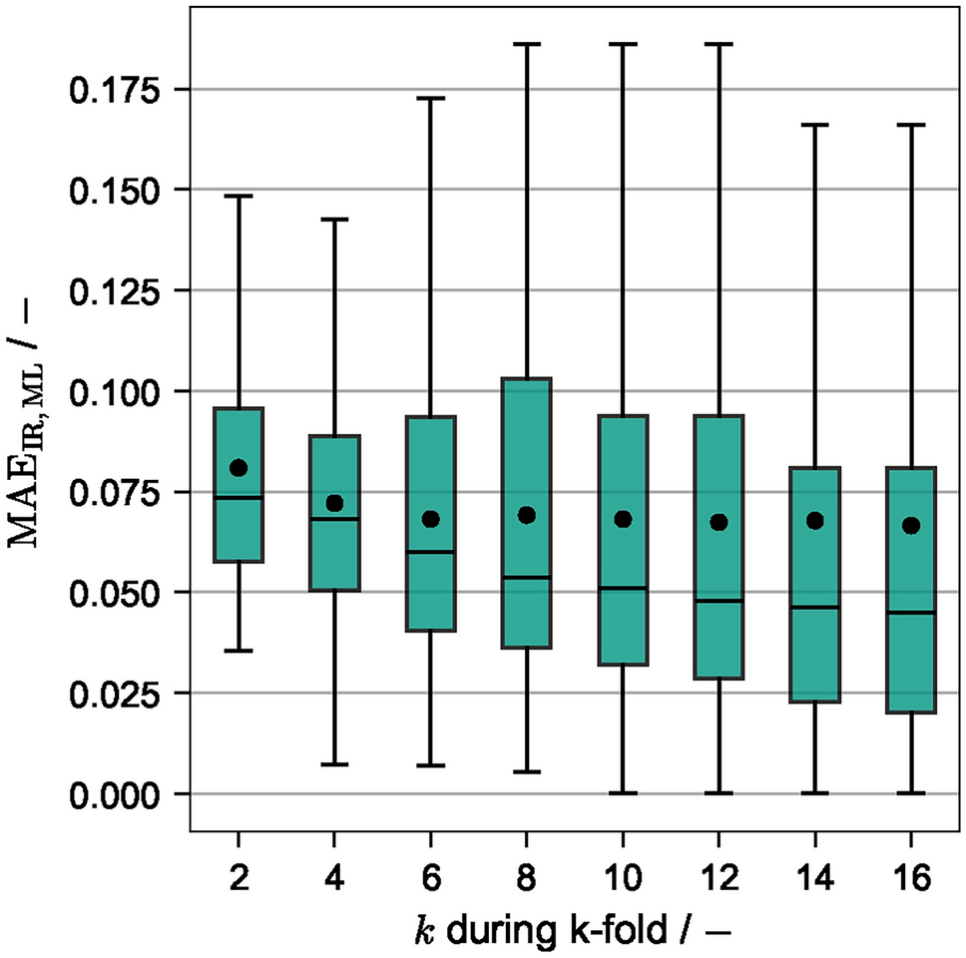
$\mathrm {MAE_{IR,ML}}$ for varying *k* during k-fold. $k\in \{2,4,8,16\}$ corresponds to $50\,\%$, $25\,\%$, $12.5\,\%$ and $6.25\,\%$ of the data being used for testing.

To investigate the influence of the amount of training data, the parameter *k* was varied between $k\in \{2,...,16\}$. These values correspond to $\{50\,\%,..., 6.25\,\%\}$ of the data being used for testing, i.e. higher k values result in less data reserved for the test set and more data being used for training. The results are given in Fig. 3 as a box plot. The box spans from the first (Q1) to the third quartile (Q3) and contains the central $50\,\%$ of the data, while the whiskers extend to the farthest data point lying within 1.5 times the inter-quartile range (IQR). The median value is indicated by a horizontal line and the mean value by a point inside the box.

Increasing *k*, i.e. increasing the amount of data being used for training versus testing, lowers both the mean and median $\text{MAE}$ across all $N\times k$ folds. However, even for $k=2$, i.e. only $50\,\%$ of the data being used for training, an $\text{MAE}<0.1$ is achieved in the majority of folds. This shows that the linear regression model is robust with respect to the required amount of training data and is not prone to overfitting. The span of the whiskers are quite large, which means that the $\text{MAE}$ exceeds 0.1 in some folds and predictions are less accurate than the IR-based method. However, these cases are retraced to extreme test/train splits, like e.g. only training for small $\text{DS}$, while predicting only large $\text{DS}$ values. Please note that this is only due to the applied cross validation with randomized test/train splits and will not be relevant for production models, i.e. routine evaluation. Here, either all or sensibly selected data is used for training, effectively eliminating these extreme cases.

#### Influence of wavenumeber ranges and feature selection

**Fig. 4:**
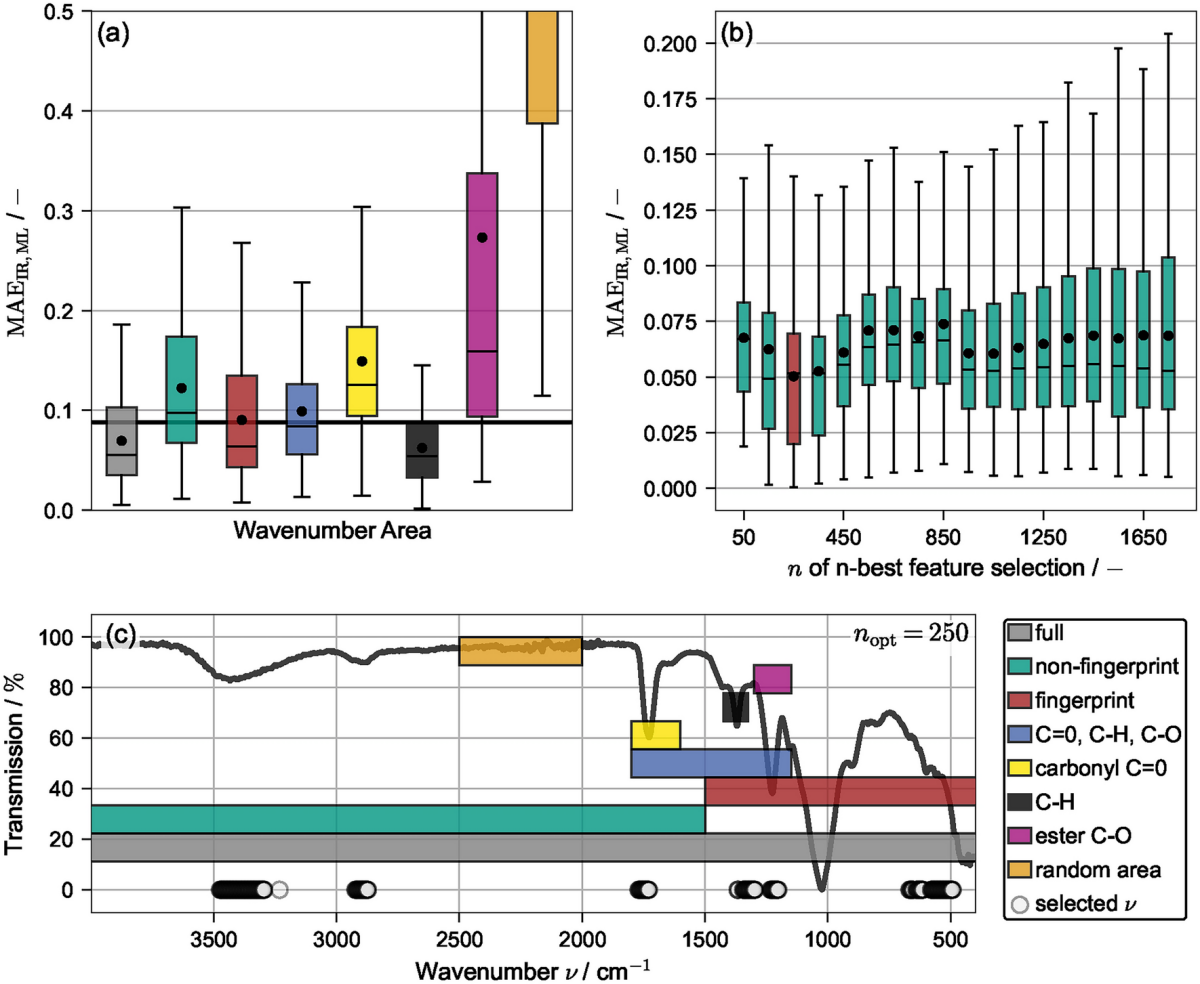
(**a**) Box-plots of $\mathrm {MAE_{IR,ML}}$ for varying wavenumber ranges viszualized in (**c**). (**b**) Box-plots of $\mathrm {MAE_{IR,ML}}$ for varying *n* of n-best feature selection. The optimum value ($n=250$) is colored in red and the selected wavelengths are visualized in (**c**).

To evaluate if and how different parts of the spectrum hold more or less information relevant for model training, model training was performed on specific wavenumber ranges, while any data outside the specific range was omitted. Fig. 4c visualizes the investigated ranges together with an exemplary spectrum: A non-fingerprint region is defined for $\nu >1500\,\text{cm}^{-1}$ and a fingerprint region for $\nu <1500\,\text{cm}^{-1}$. Additionally, peak specific ranges for ${\hbox {C}=\hbox {O}}$ ($1600\,\text{cm}^{-1}<\nu <1800\,\text{cm}^{-1}$), ${\hbox {C}-\hbox {H}}$ ($1325\,\text{cm}^{-1}<\nu <1425\,\text{cm}^{-1}$), ${\hbox {C}-\hbox {O}}$ ($1150\,\text{cm}^{-1}<\nu <1300\,\text{cm}^{-1}$) and a combined range ${\hbox {C}=\hbox {O}}$, ${\hbox {C}-\hbox {H}}$, ${\hbox {C}-\hbox {O}}$ ($1150\,\text{cm}^{-1}<\nu <1800\,\text{cm}^{-1}$) are defined. For reference, both the full range and a randomly selected area with no apparent information ($2000\,\text{cm}^{-1}<\nu <2500\,\text{cm}^{-1}$) are investigated. For each area, a repeated CV was performed with $N=1000$ and $k=8$. The resulting $\text{MAE}$ values are given as box-plot in Fig. 4a and compared to the integration-based result from above indicted by a horizontal line.

As expected, the randomly selected area was not able to yield reliable predictions of the $\text{DS}$. Similarly, predicting the $\text{DS}$ solely based on the ${\hbox {C}=\hbox {O}}$ or ${\hbox {C}-\hbox {O}}$ peaks produced large deviations and is less accurate than the integration-based method. The best predictions were obtained for the full and ${\hbox {C}-\hbox {H}}$ range, followed by the fingerprint region. These results are surprising in the sense that Wolfs et al.^27^ reported highest accuracy for evaluation of the ${\hbox {C}=\hbox {O}}$, followed by the ${\hbox {C}-\hbox {O}}$ and lastly the ${\hbox {C}-\hbox {H}}$ peak. These differences can be attributed to the different model architecture used, with the ${\hbox {C}-\hbox {H}}$ peak being more suited towards a linear regression. Fig. 4a highlights the counter-intuitive fact that manually selecting specific wavenumber ranges, a routinely applied procedure in academia and industry, does not necessarily improve model accuracy. In fact, it might lead to drastically worse predictions.

However, the search for the region that contains most information and yields the best predictions can also be performed automatically via an n-best feature selection algorithm described in the methods section. A parameter study was performed varying *n* between $n\in \left[ 50,1750\right]$ in steps of 100 performing a repeated CV for each with $N=1000$ and $k=8$. The resulting $\text{MAE}$ values are shown as box plots in Fig. 4b. The full data set contains 1750 features and is shown on the far right. Reducing the number of features reduces both the $\text{MAE}$ and its spread until $n=950$, while further reduction initially increases the prediction error. Reducing *n* further yields a global minimum at $n_{\text{opt}}=250$, where $\mathrm {MAE_{opt}}=0.052$ is achieved. This represents a significant increase in prediction accuracy compared to the integration-based evaluation method. Increasing prediction accuracy by limiting the amount of features, i.e. information, seems counter-intuitive, however, this can be explained by a reduced amount of overfitting. The initially present 1750 features contain plenty of wavenumbers that do not store information on the $\text{DS}$. Excluding these from training makes the resulting models more robust and more accurate on unseen data. Vividly, the model is forced to learn the underlying relationships rather than experiment-specific random deviations. Fig. 4c shows the selected wavenumbers as scattered points at $T=0$. Besides the expected peaks for ${\hbox {C}=\hbox {O}}$, ${\hbox {C}-\hbox {H}}$ and ${\hbox {C}-\hbox {O}}$, the model also evaluates in the range $3250\,\text{cm}^{-1}<\nu <3500\,\text{cm}^{-1}$, $2850\,\text{cm}^{-1}<\nu <2950\,\text{cm}^{-1}$ and, most surprisingly, in the range $500\,\text{cm}^{-1}<\nu <650\,\text{cm}^{-1}$ of the fingerprint region. For conventional, i.e. manual ATR-FTIR characterization of organic and polymeric compounds (including cellulose derivatives), especially vibrational bands at higher wavenumbers ($\ge$ 1500 $\hbox {cm}^{-1}$) outside the fingerprint region are essential. Particularly, the finger print region is not frequently examined in data evaluation due to its complexity. The ML approach thus seems to provide benefits for data analysis a researcher would not easily identify. Hence, not only did the n-best feature selection increase the overall prediction accuracy, it simultaneously returned relevant information about where information on the $\text{DS}$ is stored in the IR spectrum. It should be noted that only raw IR data was used for model training and the feature select algorithm identified the chemically relevant areas without additional mechanistic information or guidance.

### Application to other data sets

**Fig. 5:**
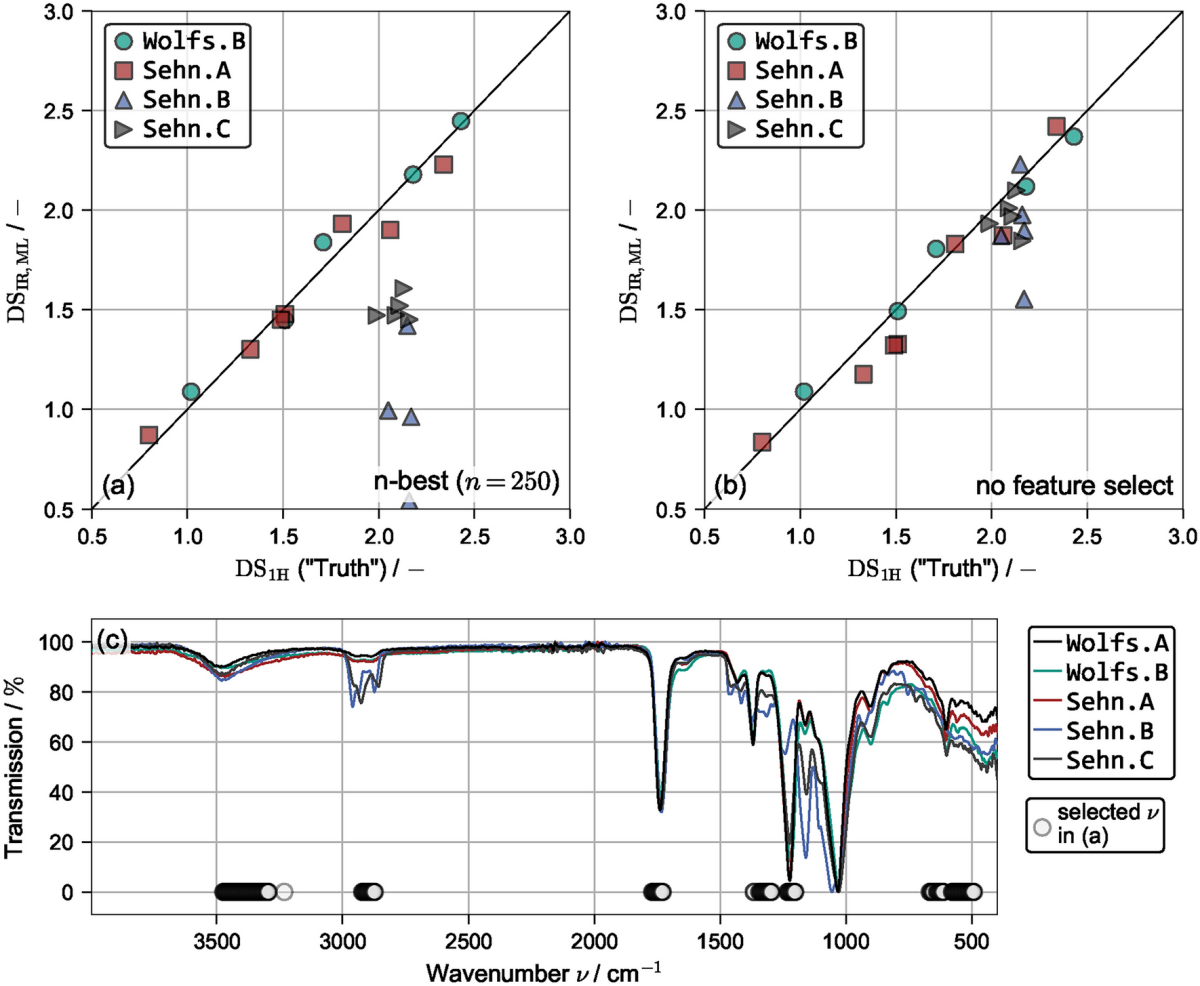
$\mathrm {DS_{1H}}$ versus $\mathrm {DS_{IR,ML}}$ values for the Wolfs.B, Sehn.A, Sehn.B and Sehn.C data set with applied n-best feature select ($n=250$) (**a**) and without feature selection (**b**). All models were trained on the full Wolfs.A data set. Exemplary IR spectra at $\mathrm {DS_{1H}}\approx 2$.

To assess the applicability of the ML evaluation method, i.e. the extrapolation to other data sets, two linear regression models were trained on the entire Wolfs.A data set. No cross validation is required as all points are included in training. One model is trained on the optimum amount of features found in the previous section ($n=250$) and one is trained on the entire range of wavelengths. Both models are used to predict the $\mathrm {DS_{IR,ML}}$ for samples contained in the Wolfs.B, Sehn.A, Sehn.B and Sehn.C data sets, which are compared to the respective $\mathrm {DS_{1H}}$ values. Fig. 5a shows the results for the model with feature selection and Fig. 5b for the one without. The resulting $\text{MAE}$ and $\text{MRE}$ values are given in Tab. 2.

**Table 2:** $\text{MAE}$ and $\text{MRE}$ values for the Wolfs.B, Sehn.A, Sehn.B and Sehn.C data set with applied n-best feature select ($n=250$) (left) and without feature selection (right). All models were trained on the full Wolfs.A data set.

Name	Feature Select ($n=250$)		No Feature Select	
	$\text{MAE}\,[-]$	$\text{MRE}\,[\%]$	$\text{MAE}\,[-]$	$\text{MRE}\,[\%]$
Wolfs.B	0.056	3.82	0.061	3.76
Sehn.A	0.080	5.00	0.118	7.58
Sehn.B	1.281	59.73	0.266	12.37
Sehn.C	0.592	28.19	0.125	5.88

Both Wolfs.B and Sehn.A are predicted with a high accuracy, demonstrating that the MLR model is robust and can be applied to IR data of different experimenters (Wolfs.B) and even different synthesis routes (Sehn.A). Additionally, the selected features seem to be universal for CA, as using the optimized model with feature select results in overall lower errors. These results show that routine evaluations of the $\text{DS}$ of CAs can be performed without the need for laborious and time-consuming ^1^H or even ^31^P NMR measurements and without the need for manual peak integration and evaluation of IR data as was done by Wolfs et al.^27^. It should be noted that all IR data was measured on the same instrument and future studies should include the influence of instrument-specific deviations on the robustness of the evaluation. However, the applied normalization in Eq. 1 alleviates this effect as long as the relative intensities between wavenumbers stay similar.

Applying the trained model to Sehn.B and Sehn.C, i.e. to other cellulose esters than CA, should be regarded as a large extrapolation task, as no IR data of different CEs was included during training. However, Fig. 5b shows that when the MLR model has access to all wavenumbers it is indeed capable of predicting the $\text{DS}$ to an accuracy of $\approx 0.1-0.2$, with only cellulose hexanoate of Sehn.B being predicted poorly ($\Delta \text{DS}=0.62$). This shows that to some degree, the $\text{DS}$ influences the IR spectra of different CEs quite similarly and that a machine learning model is capable of learning these effects. However, the model with feature select shown in Fig. 5a is unable to predict other esters, with the $\text{MAE}$ even exceeding 1 for Sehn.B. To discuss this drastically different behavior, exemplary IR spectra of all data sets are visualized in Fig. 5c. The samples closest to $\mathrm {DS_{1H}}=2$ were selected, meaning that for the linear model to yield similar predictions, all curves need to be similar. Again the selected wavenumbers ($n=250$) are indicated with scattered points. In general, all spectra are similar, especially in the expected ranges for the ${\hbox {C}=\hbox {O}}$, ${\hbox {C}-\hbox {H}}$ and ${\hbox {C}-\hbox {O}}$ peaks, but also at $\nu \approx 3500\,\text{cm}^{-1}$. However, both the peak at $\nu \approx 2900\,\text{cm}^{-1}$ and the fingerprint region differ quite strongly for different CEs. As these regions contain many of the selected wavenumbers, it is not surprising that the reduced model has difficulties in predicting the $\text{DS}$. By not applying any feature selection, these areas are less important and prediction accuracy is increased for these different CEs.

It should be noted that the objective of the trained model was not to predict the $\text{DS}$ of CEs other than CA and that it lacked the required data to accurately do so. Nevertheless, Fig. 5c suggests that this may indeed be possible with enough relevant training data. Two adjusted model structures can be conceptualized: First, a combined model with two output parameters—one being the $\text{DS}$ and one being e.g. the chain length of the ester or smiles string of the substituent—could be trained. Secondly, it is sensible to train multiple ester-specific models for prediction of the $\text{DS}$ and combine them with a superordinate classification model, which predicts the type of ester and hence, which model to apply.

### A note on model architecture

This work did not apply other model architectures than the MLR, although in the contemporary machine learning trend, neural networks (NN) are often displayed as somehow superior. It is important to stress that NN are only one of many different model architectures and that all have specific benefits and downsides. The MLR achieved a prediction accuracy close to ^1^H-based evaluation, especially after feature selection. Considering that $\mathrm {DS_{1H}}$ values also contain experimental errors, it is argued that a NN has not much room for improvement and even if slightly lower numbers were obtained, this might not represent an improvement with respect to the true $\text{DS}$. Therefore, if multiple models do not differ significantly in accuracy, one should always choose the one with fewer hyper-parameters. For a NN to perform well, the number of layers, number of nodes in each layer and activation functions have to be chosen correctly, i.e. optimized with respect to the training data. Otherwise NN are highly prone to overfitting and are known for their poor extrapolation capabilities. A previous study showed how this can be done directly during training^47^, however, the increased work and complexity did not seem to be justified in this context. Additionally, a NN is a black box without any apparent meaning to the individual weights, while the magnitude of the specific weights of the MLR herein directly corresponds to the relative importance of a specific wavenumber.

## Conclusions

This work demonstrates that a simple multiple linear regression model can be trained based on a small data set of raw IR spectra of CAs and corresponding $\text{DS}$ values, which is capable of predicting the $\text{DS}$ of unseen spectra with high accuracy, i.e. mean absolute errors of $<0.1$. This approach does neither require any manual IR data processing like baseline corrections nor manual evaluations like integration or calibration and is therefore highly interesting for fast and unbiased routine analyses in industry and academia. Applying a feature selection algorithm did not only result in lower prediction errors, but simultaneously provided insights into the wavenumber areas, where information on the $\text{DS}$ is stored. The fingerprint region was identified as relevant, which would commonly be neglected when evaluating manually. This methodology can generally be applied to other spectroscopic measurements, as long as information on the DS is stored within. The trained model was able to predict the $\text{DS}$ based on raw IR data of other experimenters applying a different synthesis route, demonstrating the robustness of the approach. It should be emphasized that the data used in this study was not specifically generated to train regression models. When training a model for production, i.e. routine evaluations of $\text{DS}$, more and most importantly evenly spaced data should be recorded to build an even more reliable model without bias towards certain $\text{DS}$ ranges.

The trained model was applied to different CEs and achieved overall good prediction accuracy. This is surprising considering the degree of extrapolation, as only CAs were used during training. A closer inspection of the IR spectra suggests that with enough data, not only $\text{DS}$ prediction, but also ester identification should become feasible. Future work should focus on applying design of experiment techniques to systematically extend the data set to different CEs. With this data, the proposed model architectures should be tested, to obtain a more generalized evaluation model for cellulose esters. If necessary, the models could be extended with other forms of input data, such as images or different spectroscopic measurements, to allow for a more differentiated prediction.

## Methods

### ^1^H integration routine

As discussed in the introduction, DS determination via ^1^H NMR is a frequently employed tool. However, the method may suffer from individual baseline correction and integration, i.e. the process depends on individual researchers and thus may include individual bias. Hence, the following step-by-step protocol for the DS determination of CA via ^1^H NMR using *MestReNova* is introduced and was used in this study to avoid such errors:

1. Reference ppm values to solvent signal (DMSO-*d*$_{6}$ = 2.50 ppm)
2. Choose *“Multipoint Baseline Correction”*
3. Apply *“Multipoint Baseline Correction”* in the following areas:
  - From 0.00 ppm to 0.50 ppm
  - From 6.00 ppm to 10.0 ppm
4. Integration of magnetic resonances in the following areas:
  - From 1.40 ppm to 2.25 ppm (acetyl signal)
  - From 2.73 ppm to 6.00 ppm (AGU signal)

### IR data normalization

Before use, the raw IR transmission spectrum of each sample *j*, denoted by ${\mathbf{T}}_{\text{raw},j}\in {\mathbb {R}}^{1\times M}$, where *M* is the number of wavenumbers, is scaled according to the min-max normalization in Eq. 1. Throughout this publication, arrays are notated in bold to distinguish them from integers.

1 $$\begin{aligned} {\mathbf{T}}_j = \frac{{\mathbf{T}}_{\text{raw},j}-\text{min}({\mathbf{T}}_{\text{raw},j})}{\text{max}({\mathbf{T}}_{\text{raw},j})-\text{min}({\mathbf{T}}_{\text{raw},j})} \end{aligned}$$

### ML model structure and implementation

The goal of this work was to train and evaluate regression models that are able to predict the $\text{DS}$ based on a provided IR spectrum. Training was achieved using static data, so supervised learning was applied. The main success-defining factor is the chosen model architecture. In this study, one of the simplest model architectures, a multiple linear regression (MLR) model was used that is based on ordinary least squares. For any given training set, the $\text{DS}$ data is structured in a ${\mathbf{D}}\in {\mathbb {R}}^{S\times 1}$ array, while the corresponding spectroscopic data is structured in a ${\mathbf{T}}\in {\mathbb {R}}^{S\times M}$. *S* is the number of samples and *M* is the amount of wavenumbers (features), while in this case the amount of predicted values (outputs) is one (i.e. the $\text{DS}$). A MLR model ${\mathcal {M}}$ is defined by a set of weights ${\mathbf{w}}\in {\mathbb {R}}^{M\times 1}$, so that

2 $$\begin{aligned} {\mathbf{D}}_{\text{IR,ML}}= {\mathcal {M}}\left( {\mathbf{T}}\right) = {\mathbf{T}}{\mathbf{w}} \quad . \end{aligned}$$

During training, the weights are optimized according to

3 $$\begin{aligned} \min _{w} || {\mathbf{T}}{\mathbf{w}} - {\mathbf{D}}||_2^2 \end{aligned}$$

and the model was implemented with the scikit-learn^48^ class LinearRegression in Python. As $\text{DS}$ values can only be positive, the ReLU (rectified linear unit) function $f(x)=\text{max}(0,x)$ is applied to the MLR output to enforce this physical constraint. The beauty of a MLR model is that it has only one relevant hyper parameter: A constant term can be included in the features and the corresponding weights correspond to the wavenumber-specific intercepts. This option fit_intercept was set to False in this study.

### Quality metrics

The mean absolute error (MAE) between any reference ${\mathbf{D}}_{\text{ref}}$ and evaluation data set ${\mathbf{D}}_{\text{eval}}$ of size ${\mathbb {R}}^{S\times 1}$ is defined as

4 $$\begin{aligned} \text{MAE}=\frac{1}{S}\sum \limits _{i=1}^{S}\left| {\mathbf{D}}_{\text{eval},i}-{\mathbf{D}}_{\text{ref},i} \right| \end{aligned}$$

and has the same dimension as $\text{DS}$. Similarly, the mean relative error (MRE) is defined as

5 $$\begin{aligned} \text{MRE}=\frac{1}{S}\sum \limits _{i=1}^{S}\frac{\left| {\mathbf{D}}_{\text{eval},i} - {\mathbf{D}}_{\text{ref},i} \right| }{{\mathbf{D}}_{\text{ref},i}} \quad . \end{aligned}$$

### Repeated cross validation

**Fig. 6:**
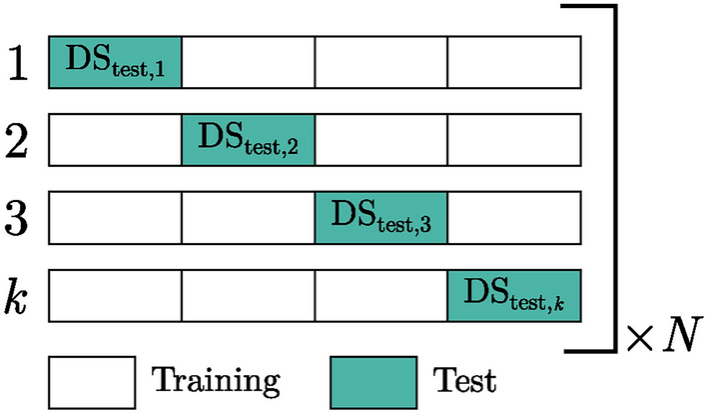
Visualization of the repeated cross validation procedure.

To quantify the predictive performance of any supervised learning model, it is important to test it on unseen data that has not been used during training. Hence, the full data set available is initially split into a train and test subset, with self-explanatory intention. However, this is problematic because the model performance, e.g. the MAE in Eq. 4, is only evaluated on the test subset. To quantify the model performance on the entire data set, a *k*-fold cross validation (CV) is routinely used that is visualized in Fig. 6. The full data set is split into *k* subsets, ensuring that every data point is exclusively contained in one subset. Subsequently, these subsets are used as test data, while the remaining points are used for model training. In essence, *k* individual models are trained and each is evaluated on the respective test data. Subsequently, Eq. 4 can be applied to the combined predictions of the *k* test subsets, yielding an objective metric for model accuracy. As the assignment into *k* subsets is generally done randomly, there are various possible data combinations that differ in model performance. To eliminate this stochastic behavior, the *k*-fold CV is repeated *N* times with randomized data assignment between repetitions. The RepeatedKFold class of the scikit-learn^48^ package was used.

### Feature select

The idea of a feature select (FS) algorithm is to only train the model on the most relevant features (wavenumbers). This can generally improve model performance and make a model more robust, as noisy or non-informative features are omitted. Obviously, one hast to define what *relevant* means in this context. In this work, the SelectKBest class based on the f_regression metric of scikit-learn^48^ was used. Here, the Pearson correlation coefficient

6 $$\begin{aligned} \varvec{\rho }_i = \frac{\text{cov}\left( {\mathbf{T}}[:,i],{\mathbf{D}}\right) }{\sigma ({\mathbf{T}}[:,i])\sigma ({\mathbf{D}})}= \frac{ \dfrac{1}{S-1}\sum \limits _{j=1}^{S}({\mathbf{T}}_{j,i}-\bar{T_i})({\mathbf{D}}_{j}-{\bar{D}})}{\sqrt{\dfrac{1}{S-1}\sum \limits _{j=1}^{S}({\mathbf{T}}_{j,i}-\bar{T_i})}\,\, \sqrt{\dfrac{1}{S-1}\sum \limits _{j=1}^{S}({\mathbf{D}}_j-{\bar{D}})}} \end{aligned}$$

is calculated for each individual feature *i* that measures the linear correlation between any wavenumber and the $\text{DS}$. $\varvec{\rho }$ is a normalized measure of covariance and can take values between $-1$ and 1. Subsequently, the F-statistic

7 $$\begin{aligned} {\mathbf{F}}_i = \frac{\varvec{\rho }_i^2}{1 - \varvec{\rho }_i^2}(n-2) \end{aligned}$$

is computed, which ranks the features between 0 and 1 and therefore also ranks strong negative correlations highly. Providing an integer *n*, the algorithm selects the n-best features from ${\mathbf{F}}$, i.e. the wavenumbers with the highest F values.

## Supplementary Information

Supplementary Information.

## Data Availability

All data used in this study is available at KITopen^46^. This includes both raw ATR-FTIR spectra as well as $\text{DS}$ values based on IR integration, ^1^H NMR and ^31^P NMR. The Python scripts for reproducing the results presented in this study are publicly available in the Github repository pdhs-group/DS_IR_ML.
