# Supplementary material for: Efficient and accurate determination of the degree of substitution of cellulose acetate using ATR-FTIR spectroscopy and machine learning
Source: Sci Rep. 2025 Jan 23;15:2904. doi: 10.1038/s41598-025-86378-0 (PMC11757746; doi:10.1038/s41598-025-86378-0)
Supplement: Supplementary file 1 — Supplementary Information. [file 41598_2025_86378_MOESM1_ESM.pdf]

Supporting Information for:

Efficient and accurate determination of degree of substitution of cellulose acetate using ATR-FTIR spectroscopy and machine learning

Frank Rhein,[a,*] Timo Sehn,[b] Michael A. R. Meier[b,c,*]

[a] Institute of Mechanical Process Engineering and Mechanics (MVM), Karlsruhe Institute of Technology (KIT), Kaiserstraße 12, Karlsruhe, 76131, Germany

[b] Institute of Organic Chemistry (IOC), Karlsruhe Institute of Technology (KIT), Kaiserstraße 12, Karlsruhe, 76131, Germany

[c] Institute of Biological and Chemical Systems– Functional Molecular Systems (IBCS-FMS), Karlsruhe Institute of Technology (KIT), Hermann-von-Helmholtz-Platz 1, Eggenstein-Leopoldshafen, 76344, Germany

Contents

1 Materials 2

2 Synthetic procedures 2

3 ^{1}H NMR measurements 4

4 Instruments 4

1 Materials

Samples Wolfs.A and Wolfs.B

Microcrystalline cellulose (MCC) was purchased from Sigma-Aldrich and was dried at 100°C under high vacuum for 24 h prior to use. 1,8-Diazabicyclo[5.4.0]undec-7-ene (TCI, >98%) was distilled and stored over molecular sieves. Dimethyl sulfoxide (Acros Organics, dry and stored over molecular sieve), vinyl acetate (Acros Organics, >99%), trifluoroacetic acid (Acros Organics, 99%), and CO_2 (Air Liquide, 99.995%) were used without further purification. Deuterated solvents (DMSO-d_6 and $CDCl_3$) were purchased from Eurisotop. All other solvents were used in technical grade.

Samples Sehn.A, Sehn.B, and Sehn.C

Microcrystalline cellulose (MCC, Sigma-Aldrich) was dried under reduced pressure at 100 °C for 24 h prior to use. Dimethyl sulfoxide (DMSO, dried and stored over molecular sieves Acros Organics, >99.7%), vinyl acetate (Sigma-Aldrich, >99%), vinyl propionate (TCI, >98%), vinyl butyrate (TCI, >98%), vinyl valerate (Sigma-Aldrich, 97%), vinyl hexanoate (TCI, 99%), vinyl octanoate(TCI, >99%), tetramethylguanidine (TMG, TCI, >99%), and DMSO-d_6(Eurisotop, 99.8%), $CDCl_3$ (Eurisotop, 99.8%) were used without further purification. All other solvents were employed in technical grade.

2 Synthetic procedures

Sample preparation Wolfs.A

The synthetic procedure of cellulose acetates employed as training set for the ML model is published in reference [1]:

In a round-bottom flask, cellulose (500 mg, [monomeric unit] = 3.08 mmol) was suspended in 11 mL DMSO followed by the dropwise addition of 1,8-diazabicyclo[5.4.0]undec-7-ene (1.4 mL, 9.25 mmol, 3.0 eq. per AGU). After applying a CO_2 flow through the solution for 20 min at 40°C, a clear solution was obtained and vinyl acetate (0.5–4.5 eq. per AGU) was added dropwise. The homogeneous solution was then heated to 60°C and stirred for 4 h. Subsequently, the solution was added dropwise into 50 mL of isopropanol under vigorous stirring. The precipitate was vacuum filtrated and washed with isopropanol (2 × 20 mL), suspended in 40 mL isopropanol and heated under reflux 2× for 1–12 h, vacuum filtrated and dried. The final product was obtained as a white or yellow solid.[1]

Sample preparation Wolfs.B

The synthetic procedure of cellulose acetates used in data set Wolfs.B is published in reference [2]:

In a round-bottom flask, cellulose (2.00 g, 12.33 mmol) was suspended in 60 mL DMSO followed by the dropwise addition of 1,8-diazabicyclo[5.4.0]undec-7-ene (5.5 mL, 37.00 mmol, 3.0 eq. per anhydroglucose unit). After applying a CO_2 flow through the solution for 20 min at 40 °C, a clear solution was obtained and vinyl acetate (1.0–4.5 eq. per AGU, depending on the experiment) was added dropwise. The homogeneous solution was then heated to 60 °C and stirred for 4 h. Subsequently, the dark solution was added dropwise into 300 mL of isopropanol under vigorous stirring to precipitate cellulose acetate. It was then vacuum filtrated and washed with isopropanol (3 × 50 mL). The obtained precipitate was dried under high vacuum (0.02 mbar) for 24 h. In case of residual DMSO in the product, the material was stirred with 40 mL of isopropanol (or methanol) under reflux

for 1–12 h, vacuum filtrated and dried again. The final product was obtained as a white or yellow powdery substance.[2]

Sample preparation Sehn.A

The synthetic procedure of cellulose acetates used in data set Sehn.A is published in reference [3]:

In a microwave vial, microcrystalline cellulose (MCC, 0.10 g, 0.62mmol) was suspended in 3.33 mL of anhydrous DMSO followed by the dropwise addition of TMG (4.50 to 12.0 equiv. per AGU, depending on the experiment). Subsequently, a CO_2 atmosphere (15 bar) was applied in a pressure reactor for 30 min at 50 °C until a homogeneous solution was obtained. Vinyl acetate (4.50 to 12.0 equiv. per AGU, depending on the experiment) was then added, and the transparent reaction mixture was either conventionally heated (4h at 60 °C) or subjected to microwave irradiation (300 W,10 min, 100 – 140 °C, depending on the experiment). The desired CAs were precipitated under vigorous stirring in 60 mL of isopropanol and filtrated. In order to remove residual impurities and DMSO, the products were additionally stirred in 80 mL of isopropanol under reflux for 1 h, vacuum filtrated, and dried overnight under reduced pressure at 100 °C. The final products were obtained as white powdery solids.[3]

Sample preparation Sehn.B

The synthetic procedure of cellulose esters used in data set Sehn.B is published in reference [3]:

In a microwave vial, microcrystalline cellulose (MCC, 0.10 g, 0.62mmol) was suspended in 3.33 mL of anhydrous DMSO followed by the dropwise addition of TMG (0.93 mL, 0.86 g, 7.44 mmol, 12.0 equiv. per AGU). Subsequently, a CO_2 atmosphere (15 bar) was applied in a pressure reactor for 30 min at 50 °C until a homogeneous solution was obtained. The corresponding vinyl ester (7.44 mmol,12.0 equiv. per AGU) was then added, and the transparent reaction mixture was subjected to microwave irradiation (300 W) for 10 min at 140 °C. The desired CEs were precipitated under vigorous stirring in 60 mL of isopropanol (or isopropanol/water mixture 3:1 wt % for CH and CO) and filtrated. In order to remove residual impurities and DMSO, the products were additionally stirred in 80 mL of isopropanol under reflux for 1 h, vacuum filtrated, and dried overnight under reduced pressure at 100 °C. The final products were obtained as white powdery solids.[3]

Sample preparation Sehn.C

The synthetic procedure of cellulose esters used in data set Sehn.C is published in reference [3]:

In a microwave vial, microcrystalline cellulose (MCC, 0.10 g,0.62 mmol) was suspended in 3.33 mL of DMSO (anhydrous 99.9%) followed by the dropwise addition of TMG (0.93 mL, 0.86 g, 7.44mmol, 12.0 equiv. per AGU). After transferring the reaction mixture into a pressure reactor, a CO_2 atmosphere (15 bar) was applied for 30min at 50 °C until a transparent yellowish solution was obtained. Subsequently, vinyl acetate (0.34 mL, 0.32 g, 3.72 mmol, 6.00 equiv. per AGU) and the corresponding second vinyl ester (3.72 mmol, 6.00equiv per AGU) were added, and the transparent reaction mixture was subjected to microwave irradiation (300 W) for 10 min at 140°C. The desired CEs were precipitated under vigorous stirring in 60mL of isopropanol and filtrated. In order to remove residual impurities and DMSO, the products were additionally stirred in 80mL of isopropanol under reflux for 1 h, vacuum

filtrated and dried overnight under reduced pressure at 100 °C. The final products were obtained as white powdery solids. [3]

3 ^{1}H NMR measurements

Sample preparation

First, 10 to 15 mg of the corresponding cellulose derivative were fully dissolved in 500 µL of DMSO-d_6 or $CDCl_3$. Subsequently, if DMSO-d_6 was employed, a few dops of trifluoro acetic acid (TFA) were added to shift the water signal more downfield to avoid an overlap with magnetic resonances of the cellulose backbone. The ^{1}H NMR experiments were then conducted with the parameters described in the instrument section and evaluated according to the ^{1}H integration routine in section 3.1 of the manuscript.

4 Instruments

Infrared spectroscopy

Infrared spectra (IR) of all samples were recorded using a Bruker Alpha-p instrument with ATR technology in a range of ν = 400–4000 cm^{-1} with 24 scans per measurement and a resolution of 4 cm^{-1}. All samples were dried at 100°C and reduced pressure (30 mbar) for 12 h before the measurement to remove traces of adsorbed water from the sample.

Nuclear magnetic resonance spectroscopy

Wolfs.A

^{1}H NMR spectra were recorded using a Bruker Avance DRX instrument at 500 MHz with 16 scans and a delay time d_1 of 5 s at 298 K. The chemical shift was reported in ppm and referenced to the solvent signal of DMSO-d_6 at 2.50 ppm and $CDCl_3$ at 7.26 ppm.

Wolfs.B, Sehn.A, Sehn.B, and Sehn.C

^{1}H NMR spectra were recorded using a Bruker Ascend 400 MHz with 16 scans and a delay time d_1 of 5 seconds at 298 K. The chemical shift was reported in ppm and referenced to the solvent signal of partly deuterated DMSO-d_6 at 2.50 ppm and $CDCl_3$ at 7.26 ppm.

---

[1] J. Wolfs, F. C. M. Scheelje, O. Mateveyeva, M. A. R. Meier, *J. Polym. Sci.* **2023**, *61*, 2697–2707.
[2] J. Wolfs, M. A. R. Meier, *Green Chem.* **2021**, *23*, 4410–4420.
[3] T. Sehn, M. A. R. Meier, *Biomacromolecules* **2023**, *24*, 5255–5264.
